# TGFβ and activin A control regulatory T cells in irradiated tumors

**DOI:** 10.1186/2051-1426-3-S2-P277

**Published:** 2015-11-04

**Authors:** Claire Vanpouille-Box, Silvia Formenti, Sandra Demaria

**Affiliations:** 1 Pathology Department, NYU School of Medicine, New York, NY, USA; 2 Radiation Oncology Department, Weill Cornell Medical College, New York, NY, USA

## 

Transforming Growth Factor-beta (TGFβ) and activin A (actA) are members of the TGFβ superfamily and display overlapping biological activities, including the ability to promote the conversion of conventional CD4 T cells to regulatory T cells (Tregs). We have recently shown that in situ vaccination by local tumor irradiation is hindered by activation of latent TGFβ (Vanpouille-Box et al., Cancer Res 2015). Intriguingly, while TGFβ blockade enhanced activation of dendritic cells and T cell priming, it did increase rather than reduce intratumoral Tregs. Because there is evidence that actA and TGFβ pathway cross-regulate each other, we tested the hypothesis that upregulation of actA by RT in the presence of TGFβ blockade may be responsible for the observed Tregs increase within the tumor.

Secretion of actA by untreated and irradiated 4T1 tumor cells was quantified by ELISA. 4T1 derivatives with conditional actA knockdown (4T1^shActA^) or non-silencing control (4T1^shNS^) were engineered using inducible tetracycline plasmids and injected s.c. in BALB/c mice (day 0). *ActA* gene knockdown was induced by doxycyline at day 8. TGFβ neutralizing 1D11 or isotype control antibodies were given i.p. every other day starting on day 12. RT was delivered to the primary tumor in 6Gy fractions on five consecutive days starting at day 13. Mice were followed for tumor growth or euthanized at day 22 for analysis.

RT significantly increased actA secretion by 4T1 cells (pActA gene expression was upregulated in irradiated 4T1-tumors but was significantly higher in mice treated with 1D11 and RT+1D11. Neither in vivo 1D11 nor *ActA* gene knockdown by themselves affected tumor growth. However, each intervention significantly improved tumor control achieved by RT. TGFβ blockade in mice bearing irradiated 4T1^shActA^-tumors did not further improve tumor control but did significantly increase IFNΥ production by CD8+ T cells in response to a tumor-specific antigen. Surprisingly, inhibiting TGFβ or actA increased intratumoral Tregs. The increase in Tregs induced by RT was markedly larger in the presence of 1D11 or actA knockdown. In marked contrast, when both TGFβ and actA were inhibited Tregs numbers significantly decreased below baseline in irradiated tumors (Control: 11.6%; 1D11: 26.2%, shActA: 21%; 1D11+shActA: 13.6%; RT: 15.7%; RT+1D11: 27.5%; RT+shActA: 30.3%; RT+1D11+shActA: 7.9% of Tregs).

These data suggest a complex regulation of Tregs in the tumor by TGFβ and actA. Combined blockade of TGFβ and actA during RT may be required to optimize activation of anti-tumor T cells induced by RT.

